# Supplementary material for: MiR-501-3p Forms a Feedback Loop with FOS, MDFI, and MyoD to Regulate C2C12 Myogenesis
Source: Cells. 2019 Jun 11;8(6):573. doi: 10.3390/cells8060573 (PMC6627719; doi:10.3390/cells8060573)
Supplement: Supplementary file 1 [file cells-08-00573-s001.pdf]

Supplementary Materials

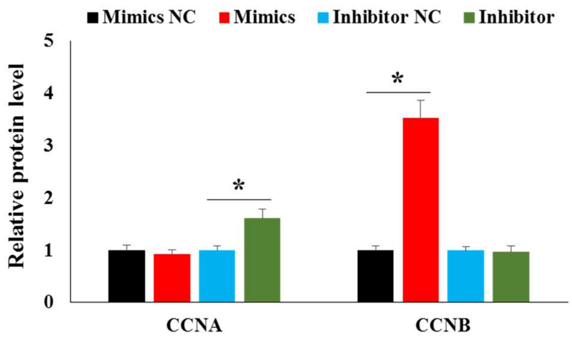

Supplementary Figure 1. WB bands quantitative results for Figure 2E.

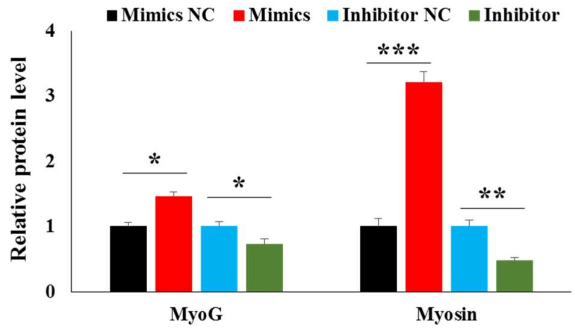

Supplementary Figure 2. WB bands quantitative results for Figure 3E

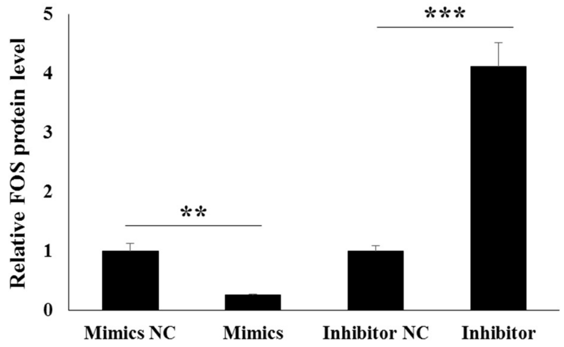

Supplementary Figure 3. WB bands quantitative results for Figure 4E.

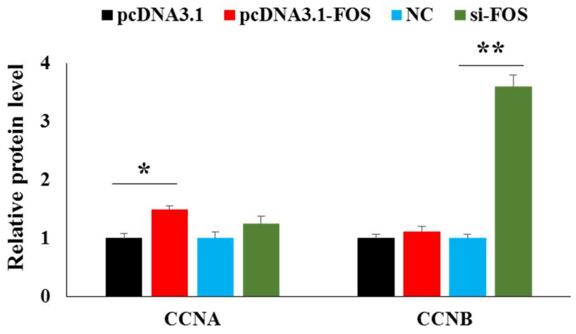

Supplementary Figure 4. WB bands quantitative results for Figure 5E.

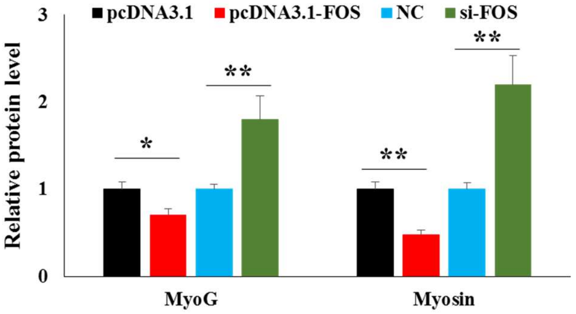

Supplementary Figure 5. WB bands quantitative results for Figure 6E.

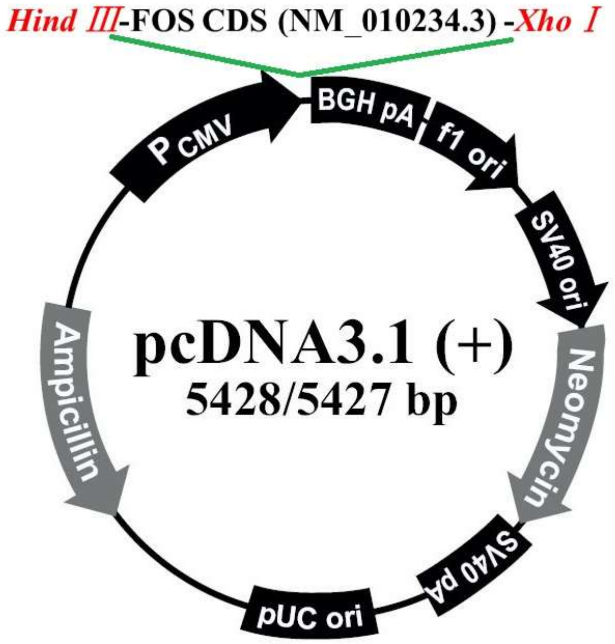

Supplementary Figure 6. pcDNA3.1-FOS information.
